# Knowledge and attitudes toward e-cigarettes as smoking cessation aids among Saudi Arabian medical students: A cross-sectional study

**DOI:** 10.18332/tid/221537

**Published:** 2026-07-11

**Authors:** Nahed S. Alharthi

**Affiliations:** 1 Department of Medical Laboratory, College of Medical Sciences, Prince Sattam bin Abdulaziz University, Al Kharj, Saudi Arabia

**Keywords:** e-cigarettes, smoking experience, smokers, medical students, family

## Abstract

**INTRODUCTION:**

E-cigarettes have garnered global attention as a potential aid for smoking cessation, yet their safety and efficacy remain subjects of ongoing debate. This cross-sectional study aimed to evaluate the knowledge and attitudes of medical students in Saudi Arabia regarding e-cigarettes as a smoking cessation tool.

**METHODS:**

A cross-sectional study was conducted between September 2023 and January 2024 among 242 medical students in a tertiary institution in Saudi Arabia. Eligible participants were currently enrolled medical students who consented to participate, while incomplete responses were excluded. Data were collected using a structured questionnaire, and associations between variables were analyzed using chi-squared tests and multivariate logistic regression models, with p<0.05 as the criterion for statistical significance.

**RESULTS:**

Among respondents, 33.2% had tried e-cigarettes, and 17.8% identified as smokers. Younger participants (≤39 years), males, and those in earlier academic years exhibited significantly better knowledge and more favorable attitudes (p<0.001). Participants with exposure to cigarette or e-cigarette smoking through family or friends, demonstrated higher knowledge scores (p<0.0072 and p<0.001, respectively). High GPA was significantly associated with better knowledge (p<0.001) and more positive attitudes (p=0.0473). At the same time, positive attitudes were also more common among students with chronic diseases (p<0.001) and those with a family history of mental disorders (p<0.001).

**CONCLUSIONS:**

Gender, academic exposure, and firsthand smoking experiences significantly shape medical students' perceptions of e-cigarettes. These findings suggest a need for targeted educational interventions to enhance evidence-based understanding and better prepare future physicians for smoking cessation counseling.

## INTRODUCTION

Tobacco use remains a major preventable cause of mortality worldwide, contributing to approximately eight million deaths each year due to smoking-related illnesses, including lung cancer, cardiovascular diseases, and chronic obstructive pulmonary disease (COPD)^1^. In Saudi Arabia, the prevalence of tobacco use remains a public health challenge, with estimates of 20% of the population^2^. Despite significant efforts by the Saudi government to combat tobacco use, including the implementation of high tobacco taxes, smoking bans in public areas, and cessation initiatives, the uptake of smoking cessation remains suboptimal^3^. These limitations are further complicated by the growing prevalence of alternative nicotine delivery systems, particularly e-cigarettes, which have gained popularity as both recreational devices and potential smoking cessation aids.

E-cigarettes are marketed as a safer alternative to combustible tobacco products, delivering nicotine via aerosol without the harmful byproducts of combustion. Some research supports their potential role as a smoking cessation aid, with randomized controlled trials in some countries demonstrating comparable or superior efficacy to traditional nicotine replacement therapies (NRTs)^4,5^. However, concerns about the safety of e-cigarettes have emerged, with evidence linking their use to respiratory illnesses, cardiovascular risks, and nicotine addiction^6^. Moreover, the dual use of e-cigarettes alongside traditional tobacco products, particularly among younger populations, has raised alarms about their potential to normalize smoking behaviors^7^. In Saudi Arabia, while e-cigarettes have gained significant popularity, research on their effectiveness as cessation tools remains limited^8,9^. This lack of local data necessitates an investigation into the perceptions and knowledge of healthcare professionals and medical students, who are central to public health advocacy and patient education.

Globally, healthcare providers are at the forefront of smoking cessation interventions, offering evidence-based advice and facilitating access to effective cessation aids. Studies have shown that healthcare providers with adequate knowledge and training are more likely to engage in smoking cessation counseling and recommend appropriate interventions^10,11^. Their knowledge and attitudes toward e-cigarettes as a cessation tool are essential in determining their ability to educate patients and implement smoking cessation strategies effectively^11^.

This study aims to assess Saudi Arabian medical students’ knowledge and attitudes toward e-cigarettes as smoking cessation aids, providing vital insights into how personal and academic factors shape future physicians’ views on e-cigarettes and revealing the urgent need for targeted, evidence-based education.

## METHODS

### Study design, sample size, study population

This cross-sectional study targeted medical students in a selected tertiary institution in Saudi Arabia. Participants were recruited using a purposive sampling approach, in which eligible medical students across different academic years were invited to participate voluntarily through institutional communication channels. Efforts were made to ensure representation across academic levels; however, no formal randomization or stratified sampling was employed. Participants were selected from different academic years to ensure diverse academic exposure. The inclusion of older students reflects delayed or non-linear medical training pathways common in the region, including second-degree entrants and returning students. A structured questionnaire-based survey was employed to gather data on participants’ demographics, academic and clinical data, as well as their knowledge and attitudes towards cigarette or e-cigarette smoking. This study was conducted over five months from September 2023 to January 2024. The sample size was determined using Raosoft, applying a 5% margin of error, a 95% confidence level, and an assumed prevalence of knowledge gaps regarding e-cigarettes as a smoking cessation tool at approximately 25%, based on the limited availability of prior data in the region. Based on this calculation, a minimum sample size of 224 participants was determined to be sufficient to achieve the study’s objectives. However, to account for potential non-responses or incomplete data, a buffer of approximately 8% was included, ensuring the recruitment target would accommodate attrition while maintaining statistical power. This increased the sample size to 242. The inclusion criteria were medical students who were currently enrolled and who consented to participate in the study. Students from non-medical disciplines and those who declined to participate or submitted incomplete responses were excluded to maintain data integrity.

### Data collection tool

The questionnaire consisted of three structured sections. The first section included 5 items assessing sociodemographic and academic characteristics (e.g. age, gender, nationality, academic year, GPA). The second section comprised 10 items capturing clinical and exposure-related variables, including BMI, presence of chronic diseases, smoking status, and exposure to cigarette or e-cigarette smoking within family or social networks. The third section included 10 items assessing knowledge and attitudes toward e-cigarettes as smoking cessation aids. These items covered domains such as perceived safety, effectiveness in smoking cessation, addiction potential, and role in clinical counseling. Responses were measured using a Likert scale and categorical formats. A full version of the questionnaire is provided in the Supplementary file.

The third section had ten questions and was scored as follows: responses ranged from strongly agree to neutral to strongly disagree. Neutral responses were scored 0, while the other options were scored 1–4 depending on correctness. The response options were Yes, No, and Not sure: 0 was assigned for Not sure, while 1 and -1 were assigned based on the correctness of each question for Yes and No responses. A knowledge score of ≥2 indicated good knowledge of e-cigarette use, while a score of <2 indicated poor knowledge. An attitude score of ≥8 indicated a positive attitude towards e-cigarette use and smoking cessation, while a score <8 indicated a negative attitude. The questionnaire’s validity was assessed through expert review, and its reliability was confirmed using Cronbach’s alpha.

### Statistical analysis

Statistical analysis was performed using GraphPad Prism version 8.0 (GraphPad Software, San Diego, California, USA). Descriptive statistics, such as frequencies and percentages, were utilized to summarize categorical variables. Knowledge scores were stratified into two categories, Poor or Good, based on understanding of e-cigarettes as smoking cessation aids. Attitudes were classified as Positive or Negative with specific reference to perceptions of e-cigarettes in smoking cessation and clinical counseling contexts. Chi-squared tests were used to evaluate associations between demographic variables and levels of knowledge and attitude categorization. To identify possible predictors of better knowledge and positive attitudes, two multiple logistic regression models were constructed, incorporating covariates from sociodemographic and academic information, as well as clinical data and smoking exposure. Statistical significance was determined at p<0.05, with all analyses performed as two-tailed tests.

## RESULTS

A total of 242 respondents were included in the analysis. Of these, 129 (53.3%) were aged ≤39 years, while 98 (40.5%) were male and 144 (59.5%) were female. The majority were Saudi nationals (234/242; 96.7%), unmarried (216/242; 89.3%), and without children (223/242; 92.1%). Most participants resided in Riyadh (212/242; 87.6%) and lived with their families (218/242; 90.1%). Academic distribution was relatively even across years, with the highest representation in the 4th year (56/242; 23.1%). Nearly half of the participants (113/242; 46.7%) reported a high GPA of 4.5-5 (Supplementary file Table 1).

Regarding clinical characteristics, 123/242 (50.8%) had a normal BMI, 51/242 (21.1%) were overweight, and 34/242 (14.0%) were underweight. Most participants reported no chronic disease (172/242; 71.4%), with asthma being the most common condition among those affected (19/242; 7.9%). In terms of smoking behavior, 43/242 (17.8%) identified as smokers, while 80/242 (33.2%) reported having ever used e-cigarettes. Among participants, 134/242 (55.6%) reported having an immediate family member who smoked (Table 1).

**Table 1:** Clinical characteristics and smoking behaviors of medical students in a Saudi Arabian Tertiary Institution, September 2023 – January 2024 (N=242)

*Variables*	*Category*	*n*	*%*
**Clinical data**			
**Weight** (kg)	<50	39	16.1
	50–60	67	27.3
	61–70	45	18.6
	71–80	62	25.6
	81–90	15	6.2
	>90	14	5.8
**Height** (cm)	<150	25	10.3
	150–160	74	30.6
	161–170	92	38
	171–180	51	21.1
**BMI** (kg/m^2^)	Underweight (<18.5)	34	14
	Normal weight (18.5–24.9)	123	50.8
	Overweight (25.0–29.9)	51	21.1
	Class I obesity (30.0–34.9)	21	8.7
	Class II obesity (35.0–39.9)	9	3.7
	Class III obesity (>40.0)	4	1.7
**Chronic disease**	None	172	71.4
	Asthma	19	7.9
	Anemia	9	3.7
	Headache	10	4.1
	Hypertension	4	1.7
	Diabetes	6	2.5
	Gastroesophageal reflux	3	1.2
	Multiple sclerosis	3	1.2
	Inflammatory bowel disease	2	0.8
	Kidney disease	1	0.4
	Other	18	7.43
**Family history of mental disorder**	No	184	76
	Yes	58	24
**Exposure to cigarette and e-cigarette smoking**			
**Smoking status**	Smoker	43	17.8
	Non-smoker	185	76.4
	Ex-smoker	14	5.8
**Immediate family member smokes**	No	107	44.4
	Yes	134	55.6
**Ever use e-cigarettes**	No	162	66,8
	Yes	80	33.2
	<Once per month	22	9.1
	Monthly	7	2.9
	Weekly	9	3.7
	Daily	23	9.5
	Not specified	180	74.4
**Current use of e-cigarettes**	No	204	84,3
	Yes	38	15.7
**Family member or friend uses e-cigarettes**	No	128	52.9
	Yes	114	47.1

BMI: body mass index.

Analysis of sociodemographic and academic factors showed that participants aged ≤39 years had a higher proportion with better knowledge (104/129; 80.6%) than those aged 40–59 years (52/77; 67.5%) and ≥60 years (12/36; 33.3%) (p<0.001). Positive attitudes were observed in 94/129 (72.9%) of younger participants, compared with 52/77 (67.5%) and 24/36 (66.7%) in the older groups, although this difference was not statistically significant (p=0.6324). Male participants demonstrated significantly more positive attitudes (92/98; 93.9%) compared to females (78/144; 54.2%) (p<0.001), although a greater proportion of females exhibited better knowledge (124/144; 86.1%) compared to males (56/98; 57.1%) (p<0.001). Participants in earlier academic years showed higher proportions of better knowledge (98/155; 63.2%) compared to those in later years (82/87; 94.3%) (p<0.001). Similarly, positive attitudes varied significantly by academic year (p<0.001). Participants with high GPA (4.5–5) demonstrated greater knowledge (79/113; 69.9%) than those with lower GPA categories, and this association was statistically significant (p <0.001). Positive attitudes were also more common among those with high GPA (71/113; 62.8%) compared to lower GPA groups (p=0.0473) (Table 2).

**Table 2:** Association between sociodemographic and academic factors with knowledge and attitudes toward e-cigarettes among medical students in Saudi Arabia (N=242)

*Factors*	*Category*	*Knowledge*		p*	*Attitude*		p*
		*Good n (%)*	*Poor n (%)*		*Positive n (%)*	*Negative n (%)*	
**Sociodemographic**							
**Age** (years)	≤39	104 (42.98)	25 (10.33)	<0.001	94 (38.84)	35 (14.46)	0.6324
	40–59	52 (21.49)	25 (10.33)		52 (21.49)	25 (10.33)	
	≥ 60	12 (4.96)	24 (9.92)		24 (9.92)	12 (4.96)	
**Gender**	Male	56 (23.14)	42 (17.36)	<0.001	92 (38.02)	6 (2.48)	<0.001
	Female	124 (51.24)	20 (8.26)		78 (32.23)	66 (27.27)	
**Nationality**	Saudi	175 (72.31)	59 (24.38)	0.4337	162 (66.94)	72 (29.75)	0.0612
	Non-Saudi	5 (2.07)	3 (1.24)		8 (3.31)	0 (0)	
**Marital status**	Single	162 (66.94)	54 (22.31)	0.0535	145 (59.92)	71 (29.34)	0.0092
	Married	18 (7.44)	6 (2.48)		23 (9.50)	1 (0.41)	
	Divorced	0 (0)	2 (0.83)		2 (0.83)	0 (0)	
**Number of children**	0	168 (69.42)	55 (22.73)	0.0342	152 (62.81)	71 (29.34)	0.185
	1	9 (3.72)	0 (0.00)		8 (3.31)	1 (0.41)	
	2	3 (1.24)	1 (0.41)		4 (1.65)	0 (0)	
	3	2 (0.83)	2 (0.83)		4 (1.65)	0 (0)	
	>3	0 (0)	2 (0.83)		2 (0.83)	0 (0)	
**Residence**	Riyadh	157 (64.88)	55 (22.73)	0.7592	143 (59.09)	69 (28.51)	0.0115
	Other city	23 (9.50)	7 (2.89)		27 (11.16)	3 (1.24)	
**Living situation**	With family	158 (65.29)	60 (24.79)	0.107	146 (60.33)	72 (29.75)	0.0035
	With friends	6 (2.48)	0 (0)		6 (2.48)	0 (0)	
	Alone	16 (6.61)	2 (0.83)		18 (7.44)	0 (0)	
**Academic**							
**Academic year**	2nd	36 (14.88)	18 (7.44)	<0.001	49 (20.25)	5 (2.07)	<0.001
	3rd	24 (9.92)	21 (8.68)		40 (16.53)	5 (2.07)	
	4th	38 (15.70)	18 (7.44)		38 (15.70)	18 (7.44)	
	5th	41 (16.94)	3 (1.24)		22 (9.09)	22 (9.09)	
	6th	41 (16.94)	2 (0.83)		22 (9.09)	21 (8.68)	
**GPA**	4.5–5	79 (32.64)	34 (14.05)	<0.001	71 (29.34)	42 (17.36)	0.0473
	3.5–4.49	91 (37.60)	17 (7.02)		80 (33.06)	28 (11.57)	
	2.5–3.49	10 (4.13)	9 (3.72)		17 (7.02)	2 (0.83)	
	<2.5	0 (0)	2 (0.83)		2 (0.83)	0 (0)	

* Statistical significance at p<0.5.

Clinical and exposure-related factors showed that participants with normal BMI had better knowledge (95/123; 77.2%) than those in other BMI categories, although this association was not statistically significant (p=0.1727). However, BMI was significantly associated with attitudes (p<0.001), with positive attitudes observed in 69/123 (56.1%) of those with normal BMI. Participants with chronic diseases showed lower proportions of better knowledge (43/70; 61.4%) compared to those without chronic diseases (129/172; 75.0%) (p=0.0014), but demonstrated more positive attitudes (63/70; 90.0%) compared to those without chronic disease (109/172; 63.4%) (p<0.001). Smoking status was strongly associated with both knowledge and attitudes. Smokers demonstrated higher proportions of better knowledge (37/43; 86.0%) compared to non-smokers (141/185; 76.2%) and ex-smokers (2/14; 14.3%) (p<0.001). Similarly, positive attitudes were more common among smokers (42/43; 97.7%) compared to non-smokers (114/185; 61.6%) (p<0.001). Participants with an immediate family member who smoked had lower proportions of better knowledge (90/134; 67.2%) compared to those without (89/107; 83.2%) (p=0.0072), but demonstrated more positive attitudes (118/134; 88.1%) compared to those without such exposure (51/107; 47.7%) (p<0.001). Participants who had ever used e-cigarettes demonstrated slightly higher levels of knowledge (62/80; 77.5%) than those who had never used (118/162; 72.8%), although this difference was not statistically significant (p=0.1221). However, positive attitudes were more common among those who had ever used e-cigarettes (47/80; 58.8%) compared to those who had not (122/162; 75.3%) (p=0.0226). Finally, participants exposed to e-cigarette use through family or friends demonstrated lower proportions of better knowledge (72/114; 63.2%) compared to those without such exposure (108/128; 84.4%) (p<0.001), but more positive attitudes (88/114; 77.2%) compared to those without exposure (82/128; 64.1%) (p=0.0257) (Table 3).

**Table 3:** Association between clinical and e-cigarette exposure factors with knowledge and attitudes among medical students in Saudi Arabia (N=242)

*Factors*	*Category*	*Knowledge*		p*	*Attitude*		p*
		*Good*	*Poor*		*Positive*	*Negative*	
**Clinical**							
**Weight** (kg)	<50	19 (7.85)	20 (8.26)	<0.001	36 (14.88)	3 (1.24)	<0.001
	50–60	67 (27.69)	0 (0.00)		50 (20.66)	17 (7.02)	
	61–70	33 (13.64)	12 (4.96)		23 (9.50)	22 (9.09)	
	71–80	38 (15.70)	24 (9.92)		42 (17.36)	20 (8.26)	
	81–90	10 (4.13)	5 (2.07)		7 (2.89)	8 (3.31)	
	>90	13 (5.37)	1 (0.41)		12 (4.96)	2 (0.83)	
**Height** (cm)	<150	19 (7.85)	6 (2.48)	0.001	19 (7.85)	6 (2.48)	0.0022
	150–160	65 (26.86)	9 (3.72)		49 (20.25)	25 (10.33)	
	161–170	68 (28.10)	24 (9.92)		56 (23.14)	36 (14.88)	
	171–180	28 (11.57)	23 (9.50)		46 (19.01)	5 (2.07)	
**BMI** (kg/m^2^)	Underweight (<18.5)	22 (9.09)	12 (4.96)	0.1727	33 (13.64)	1 (0.41)	<0.001
	Normal weight (18.5–24.9)	95 (39.26)	28 (11.57)		69 (28.51)	54 (22.31)	
	Overweight (25.0–29.9)	36 (14.88)	15 (6.20)		44 (18.18)	7 (2.89)	
	Class I obesity (30.0–34.9)	14 (5.79)	7 (2.89)		18 (7.44)	3 (1.24)	
	Class II obesity (35.0–39.9)	9 (3.72)	4 (0.00)		6 (2.48)	3 (1.24)	
	Class III obesity (>40.0)	4 (1.65)	0 (0.00)		0 (0.00)	4 (1.65)	
**Chronic disease**	No	129 (53.31)	43 (17.77)	0.0014	109 (45.04)	63 (26.03)	<0.001
	Asthma	8 (3.31)	11 (4.55)		18 (7.44)	1 (0.41)	
	Anemia	9 (3.72)	0 (0)		9 (3.72)	0 (0)	
	Headache	9 (3.72)	1 (0.41)		10	0	
	Hypertension	4 (1.65)	0 (0)		4	0	
	Diabetes	4 (1.65)	2 (0.83)		6 (2.48)	0 (0)	
	Gastroesophageal reflux	0 (0)	3 (1.24)		3 (1.24)	0 (0)	
	Multiple sclerosis	3 (1.24)	0 (0.00)		0 (0.00)	3 (1.24)	
	Inflammatory bowel disease	2 (0.83)	0 (0.00)		2 (0.83)	0 (0.00)	
	Kidney disease	0 (0.00)	1 (0.41)		1 (0.41)	0 (0.00)	
	Other	13 (5.37)	5 (2.07)		13 (5.37)	5 (2.07)	
**Family history of mental disorder**	No	140 (57.85)	44 (18.18)	1.083	116 (47.93)	68 (28.10)	<0.001
	Yes	40 (16.53)	18 (7.44)		52 (21.49)	6 (2.48)	
**Exposure to cigarette and e-cigarette smoking**							
**Smoking status**	Smoker	37 (15.29)	6 (2.48)	<0.001	42 (17.36)	1 (0.41)	<0.001
	Non-smoker	141 (58.26)	44 (18.18)		114 (47.11)	71 (29.34)	
	Ex-smoker	2 (0.83)	12 (4.96)		14 (5.79)	0 (0.00)	
**Immediate family member smokes**	No	89 (36.78)	19 (7.85)	0.0072	51 (21.07)	57 (23.55)	<0.001
	Yes	90 (37.19)	44 (18.18)		118 (48.76)	16 (6.61)	
**Ever use e-cigarettes**	No	118 (48.76)	44 (18.18)	0.1221	122 (50.41)	40 (16.53)	0.0226
	Yes	62 (25.62)	18 (7.44)		47 (19.42)	33 (13.64)	
	<Once per month	17 (7.02)	5 (2.07)		15 (6.20)	7 (2.89)	
	Monthly	5 (2.07)	2 (0.83)		7 (2.89)	0 (0.00)	
	Weekly	9 (3.72)	0 (0.00)		9 (3.72)	0 (0.00)	
	Daily	22 (9.09)	1 (0.41)		17 (7.02)	6 (2.48)	
	Not specified	129 (53.31)	51 (21.07)		121 (50.00)	59 (24.38)	
**Current use of e-cigarettes**	No	148 (61.16)	56 (23.14)	0.1305	133 (54.96)	71 (29.34)	<0.001
	Yes	32 (13.22)	6 (2.48)		37 (15.29)	1 (0.41)	
**Family member or friend uses e-cigarettes**	No	108 (44.63)	20 (8.26)	<0.001	82 (33.88)	46 (19.01)	0.0257
	Yes	72 (29.75)	42 (17.36)		88 (36.36)	26 (10.74)	

BMI: body mass index.

* Statistical significance at p<0.5.

Tables 4 and 5 present predictors of knowledge and attitude using sociodemographic, academic, and clinical variables. Younger participants (≤39 years) and males were more likely to demonstrate better knowledge and positive attitudes (p<0.001 for both). Similarly, higher academic performance (GPA 4.5–5) and earlier academic years significantly predicted better knowledge scores (p=0.0033). Clinically, having a chronic disease or a family history of mental disorders was linked to more positive attitudes toward e-cigarettes (p<0.001). Smoking status and exposure to family members who smoked were significant predictors of both knowledge and attitudes.

**Table 4:** Multivariate logistic regression analysis of sociodemographic and academic predictors of knowledge and attitudes toward e-cigarettes among medical students in Saudi Arabia (N=242)

*Variables*	*Knowledge*					*Attitude*				
	*RR*	*95% CI*	*OR*	*95% CI*	p*	*RR*	*95% CI*	*OR*	*95% CI*	p*
**Age** (years) (<39 and 40–60 vs >60)	0.4135	0.2492 – 0.6232	0.1202	0.0562 – 0.2732	<0.001	0.9149	0.6802 – 1.139	0.7447	0.3411 – 1.697	0.466
	0.4936	0.2934 – 0.7644	0.2404	0.1090 – 0.5732	<0.001	0.9872	0.7226 – 1.279	0.9615	0.4320 – 2.137	0.9272
**Gender** (Male vs Female)	0.6636	0.5438 – 0.7862	0.2151	0.1195 – 0.3982	<0.001	1.733	1.492 – 2.052	12.97	5.457 – 29.36	<0.001
**Nationality** (Saudi vs Non-Saudi)	0.8357	0.4075 – 1.171	0.5619	0.1431 – 2.180	0.4337	0.6923	0.6305 – 1.030	0	0.000 – 1.054	0.0612
**Marital status** (Single/divorced vs Married)	1.009	0.7358 – 1.216	1.037	0.4000 – 2.670	0.0157	1.421	1.169 – 1.592	11.11	1.969 – 116.7	0.0039
**Number of children** (≤2 vs >2)	0.437	0.1267 – 0.9235	0.1556	0.0293 – 0.6887	0.0162	0.6949	0.6334 to 1.145	0	0.000 – 1.667	0.1065
**Residence** (Riyadh vs Other city)	1.035	0.7903 – 1.227	1.151	0.4664 – 3.050	0.7592	1.334	1.087 – 1.522	4.343	1.328 – 13.96	0.0115
**Living situation** (Living alone vs Not living alone)	1.214	0.9111 – 1.385	2.927	0.7723 – 13.13	0.1427	0.6786	0.6148 – 0.8285	0	0.000 – 0.4349	0.0041
**Academic year** (Early academic years vs Final academic years)	1.491	1.313 – 1.713	9.539	3.891 – 22.81	<0.001	0.6173	0.4866 – 0.7561	0.2256	0.1290 – 0.4145	<0.001
**GPA** (High vs Lower groups)	0.619	0.3669 – 0.8883	0.2727	0.1075 – 0.6691	0.0033	1.324	1.030 – 1.511	4.404	1.082 – 19.49	0.0339

Odds ratios (ORs) derived from logistic regression models are presented; relative risks (RRs) are shown for comparative interpretation and should be interpreted cautiously.

* Statistical significance at p<0.5.

**Table 5:** Multivariate logistic regression analysis of clinical and e-cigarette exposure predictors of knowledge and attitudes toward e-cigarettes among medical students in Saudi Arabia (N=242)

*Variables*	*Knowledge*					*Attitude*				
	*RR*	*95% CI*	*OR*	*95% CI*	p*	*RR*	*95% CI*	*OR*	*95% CI*	p*
**Weight** (kg) (≤70 vs >70)	0.8506	0.7101 – 0.9945	0.5468	0.3045 – 0.9846	0.0421	0.9286	0.7702 – 1.098	0.7835	0.4416 – 1.360	0.3957
**Height** (cm) (≤160 vs >160)	1.264	1.095 – 1.463	2.742	1.426 – 5.084	0.0019	0.963	0.8059 – 1.135	0.8817	0.5019 – 1.512	0.6585
**BMI** (kg/m^2^) (Not obese vs Obese)	1.08	0.8500 – 1.262	1.387	0.5839 – 3.598	0.4685	1.006	0.7582 – 1.222	1.019	0.4802 – 2.187	0.9627
**Chronic disease** (Not diseased vs Diseased)	0.9244	0.7631 – 1.085	0.7536	0.4171 – 1.362	0.3547	1.389	1.198 – 1.602	4.239	1.988 – 9.097	<0.001
**Family history of mental disorder** (No vs Yes)	0.9064	0.7294 – 1.074	0.6984	0.3705 – 1.347	0.2787	1.422	1.218 – 1.635	5.08	2.092 – 11.67	0.001
**Smoking status** (Smoked vs Never smoked)	0.8977	0.7196 – 1.066	0.6761	0.3572 – 1.308	0.2385	1.594	1.417 – 1.810	34.88	6.028 – 358.1	<0.001
**Immediate family member smokes** (No vs Yes)	1.227	1.059 – 1.429	2.29	1.257 – 4.124	0.0072	0.5363	0.4291 – 0.6512	0.1213	0.06437 – 0.2307	<0.001
**Ever use e-cigarettes** (No vs Yes)	1.064	0.9025 – 1.230	1.284	0.6842 – 2.382	0.4346	0.7801	0.6252 – 0.9418	0.467	0.2687 – 0.8142	0.0083
**Current use of e-cigarettes** (No vs Yes)	1.161	0.9467 – 1.333	0.4955	0.2065 – 1.214	0.1305	1.493	1.302 – 1.677	19.75	3.310 – 204.5	<0.001
**Family member or friend uses e-cigarettes** (No vs Yes)	0.7485	0.6316 – 0.8709	0.3175	0.7485	<0.001	1.205	1.023 – 1.427	1.899	1.076 – 3.366	0.0257

Odds ratios (ORs) derived from logistic regression models are presented; relative risks (RRs) are shown for comparative interpretation and should be interpreted cautiously.

* Statistical significance at p<0.5.

## DISCUSSION

This study investigated the knowledge and attitudes of medical students in Saudi Arabia toward e-cigarettes as a smoking cessation aid. The study’s findings revealed the influence of demographic, academic, and clinical factors on perceptions of e-cigarettes within Saudi Arabia’s unique socio-cultural context. When compared with other studies, particularly in the same region, several patterns and divergences emerge, pointing to the need for public health interventions and medical education^12-14^.

The sociodemographic profile of participants revealed that most respondents were female, unmarried, and residing with family, characteristics typical of Saudi medical students. The predominance of female participants (59.5%) aligns with trends observed in similar studies conducted among medical students in the Middle East, where female enrollment in medical schools has been increasing^15,16^. However, studies have revealed that male students often demonstrate more positive attitudes toward e-cigarettes, as seen in studies conducted in Saudi Arabia and Bahrain^17^. In this study, male participants were more likely to demonstrate positive attitudes, which is consistent with these trends. This is also consistent with findings from studies in other regions^18^.

The findings of this study indicated a high prevalence of cigarette smoking and e-cigarette use behaviors, with 17.8% of participants identifying as smokers and 33.2% reporting having tried e-cigarettes. This prevalence is consistent with studies from other Gulf Cooperation Council (GCC) countries, where the use of e-cigarettes among young adults is increasing. For example, a study in Kuwait by Alshaibani et al.^19^ investigated the use, perceptions, and dependence on e-cigarettes among adults. Another study, carried out by Abbasi et al.^20^ in the UAE, investigating the knowledge and use of e-cigarettes among young adults, reported similar trends in e-cigarette use among young adults. The association between exposure to smoking through family members and more positive attitudes toward e-cigarettes reflects a well-documented trend wherein familial smoking behaviors influence individual perceptions^21,22^. However, this raises concerns about the normalization of e-cigarette use in familial and social contexts, which could undermine public health efforts to discourage smoking.

Interestingly, participants in earlier academic years demonstrated better knowledge scores than those in later years. This finding contrasts with studies such as Yang et al.^23^ and Ilic et al.^24^, where knowledge about smoking cessation typically increases with clinical exposure, and those in advanced years are more likely to practice smoking cessation than those in earlier academic years. A potential explanation for this discrepancy in Saudi Arabia may lie in the lack of emphasis on smoking cessation in the medical curriculum, particularly in the later years of study when the focus shifts to clinical training. Previous research in Saudi Arabia has highlighted gaps in medical education regarding tobacco use and cessation, suggesting the need for integrating structured training on smoking cessation tools, including e-cigarettes, across all years of medical education^25,26^. The need to integrate training into medical students’ academic curricula in other regions has also been reported^27^.

High GPAs were found to predict significantly higher knowledge scores among participants. The significant association between high GPAs and higher knowledge scores aligns with findings from other studies linking academic performance to greater health awareness and engagement with evidence-based information^28^. This suggests that high-performing students may be more proactive in seeking out reliable health information, which translates to a better understanding of complex topics such as e-cigarettes and smoking cessation. These students could serve as peer educators to disseminate accurate information about e-cigarettes and smoking cessation to their peers, thereby addressing broader knowledge gaps.

Participants with firsthand exposure to cigarette or e-cigarette smoking, either personally or through family members, demonstrated better knowledge and more positive attitudes. This aligns with findings from recent studies that report individuals with direct exposure to smoking behaviors are more likely to engage with and understand cessation tools^27,29^. However, the positive attitudes among daily e-smokers in this study warrant caution, as they may reflect a biased perception of e-cigarettes as safe alternatives rather than a critical evaluation of their risks and benefits^30^. The increasing acceptance of e-cigarette use among youth and young adults is a growing global concern. These findings highlight the necessity for targeted educational initiatives to correct misconceptions and enhance evidence-based understanding.

These findings suggest potential relationships between demographic, academic, and experiential factors and perceptions of e-cigarettes, rather than definitive causal effects. Although bivariate analyses suggested differences in knowledge by BMI category, these associations were not retained in multivariate models, indicating that BMI was not an independent predictor after adjusting for confounders. Conversely, individuals with chronic diseases or a family history of mental disorders demonstrated more positive attitudes, potentially indicating a perception of e-cigarettes as less harmful or as viable alternatives to traditional smoking. These findings align with research suggesting that vulnerable populations, including those with chronic conditions, may view e-cigarettes as a safer choice despite limited evidence to support this assumption^31,32^.

### Limitations

This study has several limitations. Its cross-sectional design precludes causal inference, and reliance on self-reported data may introduce recall or social desirability bias. Furthermore, the single-institution setting may limit the generalizability of the findings to other populations. The use of predefined scoring thresholds, while informed by expert review, may not fully capture the complexity of knowledge and attitudes toward e-cigarettes. Additionally, the use of self-reported measures and predefined scoring thresholds for knowledge and attitudes may introduce misclassification bias, potentially affecting the accuracy of categorization.

Globally, the role of healthcare providers in smoking cessation is well established, with studies emphasizing the importance of accurate knowledge and attitudes toward cessation tools^10,33,34^. However, this study reveals gaps in knowledge among Saudi medical students, particularly regarding e-cigarettes, which could undermine their ability to provide evidence-based counseling in the future. Previous studies in Saudi Arabia have similarly reported deficiencies in training related to tobacco cessation, indicating a need for curriculum reform^35,36^. The integration of a comprehensive education on e-cigarettes and smoking cessation into medical training will better equip future healthcare providers to address the evolving landscape of smoking and smoking cessation tools.

## CONCLUSIONS

This study highlights variations in knowledge and attitudes toward e-cigarettes as smoking cessation aids among medical students in Saudi Arabia, influenced by demographic, academic, and experiential factors. These findings suggest the potential value of enhancing educational content related to e-cigarettes and smoking cessation within medical curricula. However, further longitudinal, multi-center studies are needed to better understand how these perceptions may influence future clinical practice.

## Supplementary Material



## Data Availability

The data supporting this research are available from the author on reasonable request.
