# Supplementary material for: Knowledge and attitudes toward e-cigarettes as smoking cessation aids among Saudi Arabian medical students: A cross-sectional study
Source: Tob Induc Dis. 2026 Jul 11;24:10.18332/tid/221537. doi: 10.18332/tid/221537 (PMC13358856; doi:10.18332/tid/221537)
Supplement: Supplementary file 1 [file TID-24-108-s1.pdf]

QUESTIONNAIRE

Knowledge and Attitudes Toward E-Cigarettes Among Medical Students

Instructions:
Please complete the following questionnaire. All responses are confidential and for research purposes only. Select the most appropriate answer for each question.

Participant ID: ________
Timestamp: ______________

Section A: Sociodemographic and Academic Information

SN	Question	Response Options
1	Age (العمر)	☐ ≤39 years ☐ 40–59 years ☐ ≥60 years
2	Gender (الجنس)	☐ Male ☐ Female
3	Nationality (الجنسية)	☐ Saudi ☐ Non-Saudi
4	Marital Status (الحالة الاجتماعية)	☐ Single ☐ Married ☐ Divorced
5	Do you have children? (هل لديك أطفال؟)	☐ No ☐ Yes → If yes, how many? ☐ 1 ☐ 2 ☐ 3 ☐ >3
6	Residence Place (مكان الإقامة)	☐ Riyadh ☐ Other city
7	Living Situation (حالة المعيشة)	☐ With family ☐ With friends ☐ Alone
8	Academic Year (السنة الدراسية)	☐ 2nd year ☐ 3rd year ☐ 4th year ☐ 5th year ☐ 6th year
9	GPA (المعدل التراكمي)	☐ 4.5–5.0 ☐ 3.5–4.49 ☐ 2.5–3.49 ☐ <2.5

Section B: Clinical Characteristics and Smoking Exposure

SN	Question	Response Options
10	Weight (الوزن)	☐ <50 kg ☐ 50–60 kg ☐ 61–70 kg ☐ 71–80 kg ☐ 81–90 kg ☐ >90 kg
11	Height (الطول)	☐ <150 cm ☐ 150–160 cm ☐ 161–170 cm ☐ 171–180 cm
12	BMI Class (مؤشر كتلة الجسم)	☐ Underweight (<18.5) ☐ Normal (18.5–24.9) ☐ Overweight (25–29.9) ☐ Obese (≥30)
13	Do you have any chronic disease? (هل لديك أي أمراض مزمنة؟)	☐ No ☐ Yes

© 2026 Alharthi N.S. DOI:10.18332/TID/221537

14	If yes, specify the disease (ما هو المرض؟)	☐ Asthma ☐ Diabetes ☐ Hypertension ☐ Anemia ☐ Gastrointestinal disease ☐ Neurological disease ☐ Other: __________
15	Family history of mental disorder (تاريخ عائلي من اضطراب نفسي)	☐ No ☐ Yes
16	Smoking Status (حالة التدخين)	☐ Smoker ☐ Non-smoker ☐ Ex-smoker
17	Immediate family member smokes? (هل أحد أفراد العائلة يدخن؟)	☐ No ☐ Yes
18	Have you ever smoked traditional cigarettes? (هل سبق لك استخدام السجائر العادية؟)	☐ No ☐ Yes
19	If yes, how often? (كم مرة؟)	☐ Once ☐ Occasionally ☐ Regularly
20	Have you ever used e-cigarettes? (هل سبق لك استخدام السجائر الإلكترونية؟)	☐ No ☐ Yes
21	Current e-cigarette use (مدخن حالياً للسجائر الإلكترونية)	☐ No ☐ Yes
22	Family member or friend uses e-cigarettes? (هل أحد أفراد العائلة أو الأصدقاء يستخدم السجائر الإلكترونية؟)	☐ No ☐ Yes

Section C: Knowledge and Attitudes Toward E-Cigarettes

Response Scale:
☐ Strongly Agree ☐ Agree ☐ Neutral ☐ Disagree ☐ Strongly Disagree

#	Question	Arabic Translation
23	E-cigarettes are approved by the FDA for smoking cessation	هل تمت الموافقة على السجائر الإلكترونية من قبل هيئة الغذاء والدواء كوسيلة للإقلاع عن التدخين؟
24	E-cigarettes reduce the risk of cancer compared to traditional cigarettes	هل تقلل السجائر الإلكترونية من خطر الإصابة بالسرطان مقارنة بالسجائر التقليدية؟
25	E-cigarettes are helpful aids for smoking cessation	هل تعتبر السجائر الإلكترونية وسيلة فعالة للإقلاع عن التدخين؟
26	I would recommend e-cigarettes to patients for smoking cessation	هل ستنصح المرضى باستخدام السجائر الإلكترونية للإقلاع عن التدخين؟
27	Despite uncertainties, e-cigarettes are better than tobacco products	رغم عدم وضوح بعض الجوانب، هل السجائر الإلكترونية أفضل من التبغ؟
28	E-cigarettes are addictive	هل السجائر الإلكترونية تسبب الإدمان؟

© 2026 Alharthi N.S.
DOI:10.18332/TID/221537

29	Physicians should be educated about e-cigarettes	هل يجب تثقيف الأطباء حول السجائر الإلكترونية؟
30	I feel confident discussing traditional cigarette use with patients	هل تشعر بالثقة عند مناقشة التدخين التقليدي مع المرضى؟
31	I feel confident discussing e-cigarette use with patients	هل تشعر بالثقة عند مناقشة السجائر الإلكترونية مع المرضى؟
32	I have received adequate education about e-cigarettes in medical school	هل تلقيت تعليمًا كافيًا حول السجائر الإلكترونية في الكلية؟

Supplementary Table 1: Sociodemographic and Academic Characteristics of Medical Students (n = 242) in a Saudi Arabian Tertiary Institution, September 2023 – January 2024

Variables	Category	Frequency	Percentage
**Sociodemographic**			
**Age**	≤39 years	129	53.3%
	40–59 years	77	31.8%
	≥ 60 years	36	14.9%
**Gender**	Male	98	40.5%
	Female	144	59.5%
**Nationality**	Saudi	234	96.7%
	Non-Saudi	8	3.3%
**Marital status**	Single	216	89.3%
	Married	24	9.9%
	Divorced	2	0.8%
**No. children**	0	223	92.1%
	1	9	3.7%
	2	4	1.7%
	3	4	1.7%
	>3	2	0.8%
**Residence place**	Riyadh	212	87%
	Other city	30	12.4%
**Living situation**	With family	218	90.1%
	With friends	6	2.5%

© 2026 Alharthi N.S. DOI:10.18332/TID/221537

	Alone	18	7.4%
**Academic Information**			
**Academic year**	2nd	54	22.3%
	3rd	45	18.6%
	4th	56	23.1%
	5th	44	18.2%
	6th	43	16.5%
**GPA**	4.5–5	113	46.7%
	3.5–4.49	108	44.6%
	2.5–3.49	19	7.9%
	**<2.5**	2	0.8%

© **2026 Alharthi N.S.**
